# Metabolic profiling reveals new serum signatures to discriminate lupus nephritis from systemic lupus erythematosus

**DOI:** 10.3389/fimmu.2022.967371

**Published:** 2022-08-19

**Authors:** Yamei Zhang, Lingling Gan, Jie Tang, Dan Liu, Gang Chen, Bei Xu

**Affiliations:** ^1^ Department of Clinical Laboratory, Mianyang Central Hospital, School of Medicine, University of Electronic Science and Technology of China, Mianyang, China; ^2^ Department of Pathology, Mianyang Central Hospital, School of Medicine, University of Electronic Science and Technology of China, Mianyang, China

**Keywords:** systemic lupus erythematosus, lupus nephritis, non-targeted metabonomics, high performance liquid chromatography tandem mass spectrometry, serum signatures

## Abstract

**Background:**

Lupus nephritis (LN) occurs in 50% of patients with systemic lupus erythematosus (SLE), causing considerable morbidity and even mortality. Previous studies had shown the potential of metabolic profiling in the diagnosis of SLE or LN. However, few metabonomics studies have attempted to distinguish SLE from LN based on metabolic changes. The current study was designed to find new candidate serum signatures that could differentiate LN from SLE patients using a non-targeted metabonomics method based on ultra high performance liquid chromatography tandem mass spectrometry (UPLC-MS/MS).

**Method:**

Metabolic profiling of sera obtained from 21 healthy controls, 52 SLE patients and 43 LN patients. We used SPSS 25.0 for statistical analysis. Principal component analysis (PCA), partial least squares discriminant analysis (PLS-DA) and metabolic pathway analysis were used to analyze the metabolic data.

**Results:**

Upon comparison of SLE and LN groups, 28 differential metabolites were detected, the majority of which were lipids and amino acids. Glycerolphospholipid metabolism, pentose and glucuronate interconversions and porphyrin and chlorophyll metabolism were obviously enriched in LN patients versus those with SLE. Among the 28 characteristic metabolites, five key serum metabolites including SM d34:2, DG (18:3(9Z,12Z,15Z)/20:5(5Z,8Z,11Z,14Z,17Z)/0:0), nervonic acid, Cer-NS d27:4, and PC (18:3(6Z,9Z,12Z)/18:3(6Z,9Z,12Z) performed higher diagnostic performance in discriminating LN from SLE (all AUC > 0.75). Moreover, combined analysis of neuritic acid, C1q, and CysC (AUC = 0.916) produced the best combined diagnosis.

**Conclusion:**

This study identified five serum metabolites that are potential indicators for the differential diagnosis of SLE and LN. Glycerolphospholipid metabolism may play an important role in the development of SLE to LN. The metabolites we screened can provide more references for the diagnosis of LN and more support for the pathophysiological study of SLE progressed to LN.

## Introduction

Systemic lupus erythematosus (SLE) is an autoimmune disease in which the immune system attacks its own tissues, causing widespread inflammation and tissue damage in the affected organs. Lupus nephritis (LN) is seen in 50% of SLE patients and is a major cause of morbidity and mortality (1). Renal biopsy is considered the gold standard for the diagnosis of LN (2), but for its invasive, repeated biopsy is not safe and is rarely monitored. Thus, in LN patients,

In recent years, serum or urine biomarkers, such as serum creatinine, immune related molecules, complement component C3b and anti-C1q antibodies, which are commonly used in the evaluation of renal function in patients with LN, have emerged. However, the markers are not sufficiently sensitive and specific to reflect the real-time immunopathological reactions of the renal (3, 4). Accurate diagnosis and active treatment can maintain the renal function of LN patients, delay the process of renal fibrosis, and thus delay the occurrence and development of end-stage renal disease (ESKD) (5). Therefore, new biomarkers are needed to accurately reflect the diagnostic efficacy and real-time pathophysiology during the progression from SLE to LN.

Metabolomics is the analysis of concentration profiles of low molecular weight metabolites present in biological fluids, which is a relatively new field including autoimmunity (6). The prevenient studies had shown the latent capacity of metabolic profiling in the distinguishing SLE/LN from HC, and multiple biomarkers have been identified (7–9). In comparison to healthy individuals, Yuhua Li, et al. found that ceramide, trimethylamine N-oxide, xanthine, and hydrocortisone were dramatically altered in SLE serum (8). Additionally, a panel of three metabolomics (theophylline, oxidized glutathione and capric acid) was identified as biomarkers of LN (7). In contrast, small-molecule biomarkers derived from metabolomics have been rarely studied when it comes to distinguishing SLE from LN. Until now, only two studies had tested metabolomics for identifying metabolic changes between SLE and LN (10, 11). One was the Chronic Kidney Disease Research Center of Tehran Medical University in Iran (10), but their sample size was relatively limited, and the experiment was carried out on urine samples, which the changes in urinary pH, varies of the concentrations of urine, the presence of bacteriuria or urinary infections could alter the result (12). The other one found that compared to SLE patients, the LN patients had increased serum levels of lipid metabolites (including low-density lipoprotein/very low density lipoprotein) and creatinine, and decreased levels of acetate (11). Both the two studies were used ^1^ H NMR spectroscopy to characterize the altered metabolic profiles. Together, the in-depth research on small molecule metabolites and specific metabolic pathways was relatively insufficient.

Ultra high performance liquid/gas chromatography-tandem mass spectrometry (UPL/GC-MS/MS respectively) and nuclear magnetic resonance (NMR) spectroscopy are the most widespread used analysis in metabolomics research. These methods have the potential to identify new biomarkers that had great discrimination to disease status or bioturbation (13–16). Mass spectrometry is a widely used metabolomics technology because of its high detection sensitivity, metabolome coverage and rapid data acquisition turnover. Moreover, UPLC-MS/MS is especially suitable for large-scale untargeted metabolic profiling (7). Therefore, this study aims to explore whether serum metabolomics analyzed by UPLC-MS/MS can reveal the specific characteristics of the progression from SLE to LN, so as to provide new candidate serum signatures for predicting the renal complications of SLE and understanding the metabolic pathway in the pathogenesis of LN.

## Materials and methods

### Design, setting and participants

This study had obtained the regulatory approval by the Ethics Committee of Mianyang Central Hospital. From July to December in 2021, a total of 116 subjects were enrolled in this study. The participants were categorized as three groups, healthy subjects without systemic active disease and renal history were recruited as the healthy control group (HC group), patients diagnosed with simple systemic lupus erythematosus without renal involvement who met the following inclusion criteria were treated as SLE group and those with renal involvement were treated as LN group. The HC group included 21 patients, SLE group included 52 patients and the LN group included 43 patients All subjects were females.

(1) Inclusion criteria: SLE was diagnosed based on the systemic lupus erythematosus guidelines by the *Ad Hoc* Committee of the American College of Rheumatology (ACR) (17), defined if at least 4 of the following 11 criteria are met continuously or simultaneously: the concrete details were described as previously (17). Patients with clinical and laboratory representation that satisfied the ACR lupus nephritis screening, treatment and management guidelines were defined as LN (18), including continuous proteinuria > 0.5 g/24 hour, or greater than 3 + if quantitation not perform, and/or cellular casts (red cell, hemoglobin, granular, tubular, or mixed).

(2) Exclusion criteria: Patients with cancerous tumors, primary nephrosis, diabetes, cardiovascular diseases, other rheumatic immune diseases and respiratory diseases.

### Sample collection and preparation

Whole blood (5 mL) was collected in sterile coagulation BD vacuum blood collection tubes in the morning after an overnight fast. Then gently reversed and mixed the sample for 10 times. We separated the serum by centrifuge (3000 rpm, 15 min) thirty minutes later at room temperature. Then about 10ml of randomly cleaned middle urine should be collected for the determination of urinary albumin and creatinine ratio (UACR). The clinical experiment should be accomplished within 2 hours after the serum samples were centrifuged, and the metabolomics analysis serum samples should be stored in the -80°C (19) refrigerator for standby.

### Measurement of common kidney function indicators

We used LABOSPECT008 AS automatic biochemical analyzer to detect the levels of serum urea (Urea), creatinine (SCreat), cystatin C (CysC) and complement component 1q (C1q). Urea was tested with the urease method, SCreat with the sarcosine oxidase method, CysC and C1q with transmission turbidimetry. We used SIEMENS BN II specific protein analyzer to detect the serum levels of C3, C4 by the method of immune scattering turbidimetry, EUROIMMUN Sprinter XL to detect serum anti-dsDNA level by immunofluorescence method. The estimated glomerular filtration rate (eGFR) was calculated by our research group based on the population of China using the eGFR formula developed (20), as shown below:


$$\text{eGFR}=78.64\times {\text{CysC}}^{-0.964}$$

We used a fully automated specific protein analyzer model A25 to measure the urinary albumin (UAlb) and urinary creatinine (UCreat) levels respectively by immunoturbidimetry and sarcosine oxidase technique. The UACR was calculated as below:


UACR=UAlb/UCreat

### Sample preparation for UPLC-MS/MS analysis

The serum samples were taken out from the -80°C refrigerator and slowly reconstituted on ice. The internal standard for positive and negative ion modes were prepared from the mixture of clenbuterol and chloramphenicol respectively. Then a mixture of serum samples (100 μL), internal standard (5 μL) and methanol-acetonitrile (1:1 v/v) (400 μL) was incubated at -20°C for 1 hour. The supernatant was obtained as previously described (21). After the compound was swirled and centrifuged, the resulting solutions were sterile filtered using 0.22 µm rated microfiltration membranes. The filtrate was transferred into an autosample vial, and a 5 µL aliquot were injected into the UPLC-MS/MS system for metabolomic analysis. Aliquots of all serum samples (10 µL) were pooled as part of the system adjustment and quality control (QC) process to prepare QC samples. The QC samples were treated in the same manner as the analytical samples and were introduced every 10 samples in the analytical sequence to evaluate the reliability of the large-scale metabolomics analysis (22).

### Instrumentation and conditions for UPLC-MS/MS analysis

Metabolomics analysis was performed on an ultra-performance liquid chromatography (UPLC) system (Agilent1290 Infinity II; Agilent Technologies Inc., CA, USA) connecting to a high-resolution tandem mass spectrometer (TripleTOF 5600 Plus; AB SCIEX,Framingham, MA, USA). An ACQUITY HSS T3 column (100 × 2.1 mm, i.d. 1.8 µm; Waters, Milford, USA) were equipped for reversed-phase separation. Mobile phases were solvent A (water containing 0.1% formic acid (v/v)) and solvent B (acetonitrile with 0.1% formic acid (v/v)). Separation was achieved with the following gradient program: a linear gradient of 99% A over initial-1.2 min; 99-30% A, 1.2-4.5 min; 30-1% A, 4.5-13.0 min; 1% A, 13.0-16.5 min; 1-99% A, 16.5-16.6 min; 99% A, 16.6-20.0 min. The instrumental set-up included the flow rate at 0.30 mL/min and the temperature of the column oven was at 30°C. The electrospray ionization (ESI) probe was operated in both negative (ESI−) and positive (ESI+) polarity modes. Instrument parameters included as follows: declustering potential of ± 80 V; collision energy of ± 10 V; source temperature of 550°C; curtain gas flow of 35 psi; ion source gas 1 (GS1) and GS2 of 55 psi; and accumulation time of 0.15 s.

### Data analysis of differential metabolites

Data acquisition and processing were performed with the acquisition software Analyst TF (version 1.7.1, AB SCIEX, USA). The metabolomics data processing was consists of a serial of processes, including peak selection, quality assurance, normalization, missing value interpolation, conversion and scaling (23, 24). The processed molecular weights of the metabolites (molecular weight error< 20 ppm) were confirmed, matched and annotated using a standard database, custom databases (Metlin, HMDB and ONE-MAP databases), and other integrated databases to achieve accurate metabolite characterization. Metabolic pathways associated with SLE and LN were found categorized using the KEGG database and MetaboAnalyst (25).

### Statistical analysis

We used SPSS 25.0 (International Business Machines Corp., USA) for statistical analysis. Normally distributed data were expressed as mean ± SD. The one-way analysis of variance (ANOVA) was used for comparisons among multiple groups. Independent samples T-test was used for comparisons between two groups. Non-normal distribution measurement data were expressed as median (interquartile range) [M (P25, P75)]. Comparisons of count data between SLE and LN groups using the Mann-Whitney U non-parametric test. We used the non-parametric Spearman rank correlation coefficient for correlation analysis between metabolites and clinical parameters. A P-value less than 0.05 denoted statistical significance. Potential diagnostic biomarker was evaluated by receiver-operating characteristic (ROC) curves. The area under the ROC curve (AUC) which is a widely used measure of discrimination in risk prediction models was reported and compared. We weighed and summarized the sensitivity and specificity of each variable. In terms of AUC, it represented the accuracy of the predictive model, where 1 was denoted 100% sensitivity and specificity, indicating perfect assignment, whereas an AUC of 0.5 indicated an unreliable test (grey line) (26).

The multivariate statistical analysis was performed using the software SIMCA 15.0.2 (Umetrics AB, Umea, Sweden). We performed principal component analysis (PCA) on UPLC-MS/MS data using unsupervised non target approach, allowing for the visualization of the comprehensive metabolome variation among groups and monitoring the steadiness over time of transversion (27). The differential metabolites were identified using partial least squares-discriminant analysis (PLS-DA). To avoid overfitting, the model was validated with 200 random permutation tests, model parameters R2 and Q2 were used to assess model validity and supply information about interpret-ability and predictability (28). In PLS-DA model, we selected metabolites with significant threshold (p<0.05) and folding change (FC) threshold >1.5 or<2/3, which can significantly distinguish the two groups. Based on the parameter of variable importance in the projection (*VIP* > 1), the key variables that contributed to classification could be identified (29).

Metabolomics had proposed a definition index to evaluate the reliability of annotation (30). The metabolites identified in the current study were defined as “Annotation” (Level 2), without detailed analysis and verification. This was also consistent with the formal definition of metabolite annotation and identification in the Metabolomics Standards Initiative (MSI) proposed by the Chemical Analysis Working Group (CAWG) (29).

## Results

### Population and clinical characteristics

?A3B2 tlsb .2pt?>The comparison of the clinical data including their first symptom, SLEDAI, renal pathological analysis, therapeutic drugs and laboratory measurements of patients is shown in **Table 1**. All laboratory measurements were normally or approximately normally distributed. In general, there were statistical differences among the HC, SLE and LN groups across all indicators (*P*< 0.05). Through pairwise comparison and analysis, we found that compared with SLE group, Urea, SCreat, UA, and CysC in LN group continuous increased while eGFR decreased (*P*< 0.05). Even though C1q, C3 and C4 decreased too, the differences were not significant.

**Table 1:** Clinical data of subjects (n = 116).

	SLE (n = 52)	LN (n = 43)	HC (n = 21)	*F, P*
Gender	Female	Female	Female	–
Age (years), Mean ± SD	38.83 ± 14.09	37.72 ± 12.32	49.42 ± 10.33	–
First attack/Recurrence	8/44	1/42	–	–
**First symptom**				
Malar Rash (%)	9 (17.31%)	18 (41.86%)	–	–
Oral ulcer (%)	0	3 (6.98%)	–	–
Discoid rash (%)	3 (5.77%)	3 (6.98%)	–	–
Photosensitivity (%)	0	2 (4.65%)	–	–
Arthritis (%)	14 (26.92%)	16 (37.21%)	–	–
Serositis (%)	0	3 (6.98%)	–	–
Renal dysfunction (%)	0	4 (9.30%)	–	–
Neurological derangements (%)	0	3 (6.98%)	–	–
Hematologic disorder (%)	5 (9.62%)	1 (2.33%)	–	–
**SLEDAI (score), Median (range)**	8 (2~16)	14 (5~26)	–	–
**Duration of disease (Years), Median (range)**	9 (2~18)	6 (1~12)	–	–
**Renal pathological analysis**				
Class IV (Diffuse segmental (IV-S) or global (IV-G) lupus nephritis) (%)	–	6 (13.95%)	–	–
Class V(Membranous lupus nephritis) (%)	–	3 (6.98%)	–	–
Class IV+ Class V (%)	–	3 (6.98%)	–	–
Class IV+ Class V+ Class VI(Advanced sclerosing lupus nephritis) (%)	–	1 (2.33%)	–	–
Refuse biopsy of the kidney (%)	–	30 (69.77%)	–	–
**Therapeutic drugs**				
Hydroxychloroquine (%)	52 (100%)	42 (97.67%)	–	–
Prednisolone (Steroid) (%)	36 (69.23%)	40 (95.25%)	–	–
Cyclophosphamide (%)	2 (3.85%)	12 (27.91%)	–	–
Azathioprine (%)	2 (3.85%)	1 (2.33%)	–	–
Methotrexate (%)	3 (5.77%)	3 (6.98%)	–	–
Maticophenolate (%)	3 (5.77%)	7 (16.28%)	–	–
**Laboratory measurements**				
anti-dsDNA positive (%)	6 (11.54%)	14 (32.56%)	–	–
C3 (g/L)	0.86 ± 0.22	0.81 ± 0.21	–	–
C4 (g/L)	0.15 ± 0.06	0.14 ± 0.07	–	–
Urea (mmol/L)	4.84 ± 1.63	7.10 ± 4.19^△▲^	5.13 ± 1.18	8.669,<0.001
SCreat (umol/L)	54.7 ± 13.24	69.0 ± 25.29^△▲^	53.2 ± 7.34	9.499,<0.001
eGFR. (ml/min/1.7)	78.10 ± 17.36^△^	62.31 ± 24.20^△▲^	91.13 ± 13.70	16.688,<0.001
UA (umol/L)	296.4 ± 93.63	368.4 ± 145.55^△▲^	308.0 ± 75.74	5.260,<0.001
CysC (mg/L)	1.07 ± 0.32^△^	1.50 ± 0.65^△▲^	0.88 ± 0.16	16.733,<0.001
C1q (mg/L)	173 ± 39.44^△^	162 ± 51.74^△^	234 ± 48.56	18.560,<0.001

HC: group of healthy controls; SLE: group of Systemic lupus erythematosus; LN: group of Lupus nephritis. Urea: serum urea; SCreat: serum creatinine; eGFR: estimate the glomerular filtration rate; UA: serum uric acid; CysC: serum cystatin C; C1q: serum complement component 1q; C3: component 3; C4: component 4.

^△^: Compared with HC group, P< 0.05; ^▲^: Compared with SLE group, P< 0.05.

F and P represented the statistical results of ANOVA analysis among all the study groups.

### The metabolomics data quality analysis

Metabolic profiling of 116 serum samples and 17 QC samples were acquired using the UPLC-MS/MS system in ESI+ and ESI- modes. 7527 positive ions and 5555 negative ions first-order peaks were proposed based on the MS/MS data. Data quality analysis was carried out before evaluating the differential metabolites. The stability of QC samples was confirmed by total ion chromatogram (TIC) and base peak intensity (BPI) diagrams (**Supplementary Figures 1A**–**D**). After QC correction and removing some abnormal samples, the PCA results of all samples (including QC samples) revealed that QC samples had good clustering (**Supplementary Figures 2A, B**). The results suggested the satisfactory stability and repeatability of the analysis platform. The anomaly samples were removed and differential metabolite analysis was performed by using characteristic variables with qualitative results.

### Multivariate statistical analysis of metabolites

**Figure 1:**
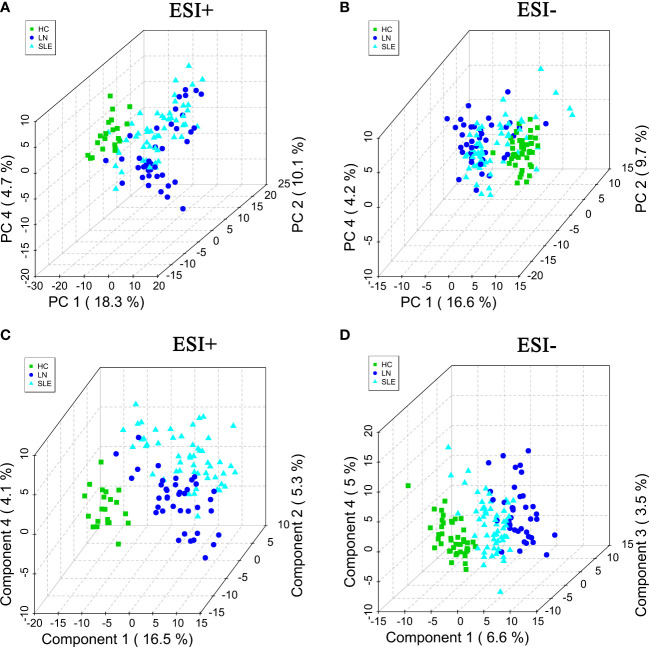
The PCA score plots in positive **(A)** and negative **(B)** ion modes among HC, SLE and LN groups. The PLS-DA score plots in positive **(C)** and negative **(D)** ion modes among HC, SLE and LN groups. PCA, principal component analysis; PLS-DA, partial least squares discriminant analysis; HC, healthy controls; SLE, Systemic lupus erythematosus; LN, Lupus nephritis.

The processed data, including the retention time, exact mass, and peak intensity, were subjected to multivariate statistical analysis. In total, 2040 metabolite features in ESI+ mode and 606 metabolite features in ESI- mode were analyzed. Subsequently, the variables were filtered based on the interquartile spacing, yielding 1216 metabolites in ESI+ mode and 454 metabolites in ESI- mode. PCA analysis was performed after data normalization. Furthermore, the raw data of all specimen about the HC, SLE and LN groups were illustrated through the score plot of the PCA. The serum components separation effect of the three groups were not very significant (**Figures 1A, B**). In order to improve the classification effect among samples, PLS-DA among the three groups were carried out. The results revealed that the three groups could be separated in the ESI+ and ESI- modes (**Figures 1C, D**), and there were significant differences between the metabolic profiles of the HC, SLE and LN groups.

### Screening of differential metabolites between groups

**Figure 2:**
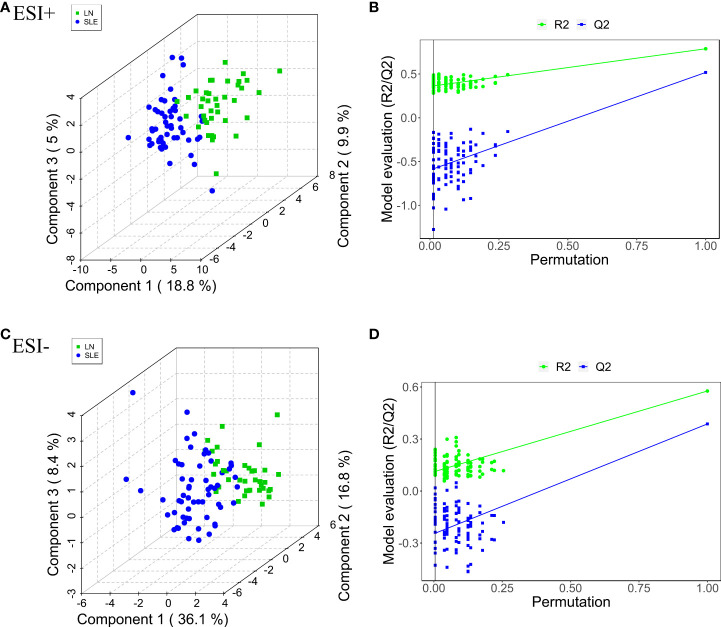
Pairwise comparison of the PLS-DA score plots in positive **(A)** and negative **(C)** ion modes and permutation test plots in positive **(B)** and negative **(D)** ion modes btween SLE and LN groups. The criterion for evaluating whether there is overfitting in the PLS-DA model is that the regression line at a blue Q2 point crosses or is less than 0 from the abscissa. PLS-DA, partial least squares-discriminant analysis; HC, healthy controls; SLE, Systemic lupus erythematosus; LN, Lupus nephritis.

The differential metabolites were screened to obtain the relevant metabolite information and analyze the difference between pairs of the groups. PLS-DA was used to filter out the signals irrelevant to the model classification, as PLS-DA features a better discriminative power than PCA. The PLS-DA results between the HC and SLE groups, HC and LN groups, and SLE and LN groups were obtained. Significant differences in classification was found between the clustering of the HC and SLE groups, HC and LN groups in both ESI+ (**Supplementary Figures 3A, B**) and ESI- (**Supplementary Figures 3C, D**) modes. Between the SLE and LN groups, the ESI+ and ESI- modes had obvious cluster separation (**Figures 2A, C**). A total of 200 random permutation tests were carried out, and both ESI+ and ESI- modes had Q2 distributions with Y- intercepts lower than zero, this indicated that no overfitting occurs here (**Figures 2B**–**D**). The results strongly certificated the dependability of the established PLS-DA model.

### Identification of potential metabolites and pathways

**Figure 3:**
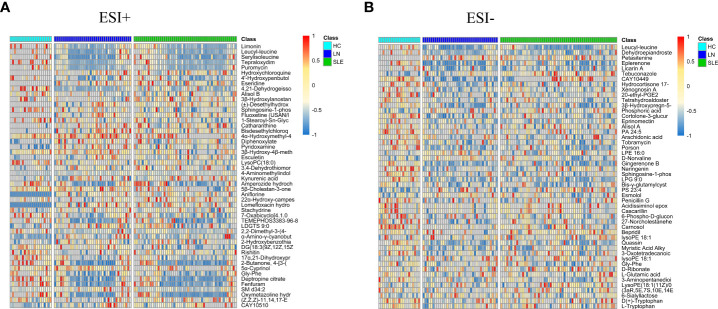
Heat maps of differential metabolites in positive **(A)** and negative **(B)** modes. The columns represent samples, the rows represent metabolites, and the relative content of the metabolites is displayed by color. The heat map shows differential metabolites among HC, SLE and LN groups. HC, healthy controls; SLE, Systemic lupus erythematosus; LN, Lupus nephritis.

On account of the analysis results of PLS-DA, with *FC* > 1.5 or < 2/3, *VIP* > 1, and *P<* 0.05 as the screening criteria, a total of 449 characteristic variables were found in the ESI+ mode and 65 characteristic variables in the ESI- mode among the HC, SLE and LN groups. The top 50 optimal characteristic variables were displayed on a heat map (**Figure 3**). Publicly available databases like KEGG, standard compounds databases, and several integrated databases were searched for qualitative identification of these characteristic variables.

Following the identification analysis, the metabolomic pathways were visualized to reveal the metabolic pathways that may be participate in the happening of LN. The acquired impact value was cumulative percentage of importance for the matched metabolite nodes, then a permutation-based *P* value was computed and corrected for multiple testing to produce a permutation-based false-discovery rate (FDR) [-log (*P*) value]. According to the pathway impact score and –log (*P*) value, the top three metabolic pathways were selected. The results identified the most critical metabolic pathways of the differential metabolites in the HC group compared with the SLE/LN groups, and the SLE group compared with the LN group. In particular, glycerophospholipid metabolism was the most obviously enriched metabolic pathway between SLE and LN group, followed by pentose and glucuronate interconversions and porphyrin and chlorophyll metabolism, please see **Table 2** for details of pathway. When HC group compared with SLE/LN groups, Arachidonic acid and glycerophospholipid metabolism had remarkable impact values and –log (*P*) values in enriched metabolic pathways (**Supplementary Table 1**).

**Table 2:** Top three significantly altered metabolic pathways between groups.

	Pathway name	KEGG.id	-log (P)	Impact	Hits
SLE vs. LN	Glycerophospholipid metabolism	hsa00564	1.904	0.339	3
	Pentose and glucuronate interconversions	hsa00040	0.696	0.141	1
	Porphyrin and chlorophyll metabolism	hsa00860	1.170	0.128	2

Impact: impact value of metabolic pathway determined by topology analysis; Hits: the number of differential metabolites matching the pathway.

### Differential metabolite analysis and diagnostic efficiencies

**Figure 4:**
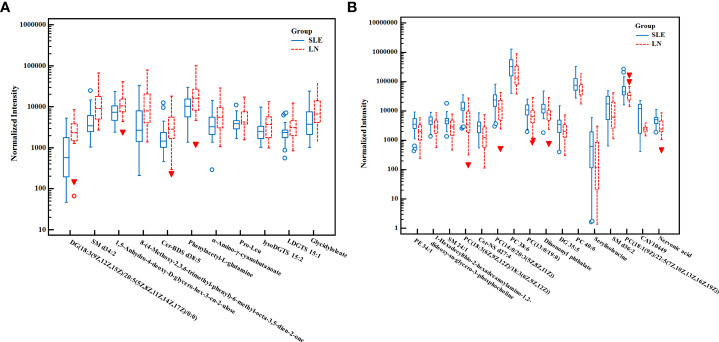
The box plot of normalized intensity peak areas of significantly increased **(A)** and decreased **(B)** metabolites in LN group when compared with SLE group. SLE, Systemic lupus erythematosus; LN, Lupus nephritis.

We used the non-parametric test to find the differential metabolites between the SLE and LN groups based on the results of *VIP* > 1.5 and *FC* > 1.5 or< 2/3 as the screening criteria. A total of 25 characteristic metabolites were found in ESI+ mode and 3 characteristic metabolites in ESI- mode. Among these 28 metabolites, 11 metabolites were significantly increased (*Z* = 2.29~4.61, *P* = 0.00~0.02), and 17 metabolites were significantly decreased in the LN group (*Z* = -4.23~-2.41, *P* = 0.00~0.02) when compared with SLE group. The 28 metabolites are listed in **Table 3** and their normalized intensity peak areas are presented in **Figure 4**.

**Table 3:** Significantly differential metabolites between SLE and LN groups.

Metabolites	FC	VIP	*Z*	*P*	Trend
DG (18:3(9Z,12Z,15Z)/20:5(5Z,8Z,11Z,14Z,17Z)/0:0)	2.46	2.45	4.47	0.00	↑
SM d34:2	2.89	2.31	4.61	0.00	↑
1,5-Anhydro-4-deoxy-D-glycero-hex-3-en-2-ulose	1.66	2.04	2.95	0.00	↑
8-(4-Methoxy-2,3,6-trimethyl-phenyl)-6-methyl-octa-3,5-dien-2-one	2.82	2.00	3.65	0.00	↑
Cer-BDS d38:5	1.85	1.90	3.33	0.00	↑
Phenylacetyl-L-glutamine	1.90	1.82	2.54	0.01	↑
α-Amino-γ-cyanobutanoate	1.70	1.77	2.29	0.02	↑
Pro-Leu	1.77	1.75	2.35	0.02	↑
lysoDGTS 15:2	1.64	1.73	2.50	0.01	↑
LDGTS 15:1	1.58	1.64	2.40	0.02	↑
Glycidyloleate	1.69	1.53	2.69	0.01	↑
PE 34:1	0.57	2.26	-3.81	0.00	↓
1-Hexadecylthio-2-hexadecanoylamino-1,2-dideoxy-sn-glycero-3-phosphocholine	0.65	2.21	-3.72	0.00	↓
SM 24:1	0.62	2.02	-3.54	0.00	↓
PC (18:3(6Z,9Z,12Z)/18:3(6Z,9Z,12Z))	0.56	2.01	-3.84	0.00	↓
Cer-NS d27:4	0.57	1.95	-3.97	0.00	↓
PC (14:0/20:3(5Z,8Z,11Z))	0.60	1.90	-3.12	0.00	↓
PC 38:6	0.60	1.88	-2.69	0.00	↓
PC (13:0/19:0)	0.64	1.86	-3.15	0.00	↓
Diisononyl phthalate	0.63	1.73	-3.62	0.00	↓
DG 35:5	0.61	1.65	-2.41	0.02	↓
PC 40:6	0.63	1.64	-3.11	0.00	↓
Serylisoleucine	0.46	1.58	-2.57	0.00	↓
SM d36:2	0.61	1.51	-2.45	0.01	↓
PC (18:1(9Z)/22:5(7Z,10Z,13Z,16Z,19Z))	0.63	1.51	-3.44	0.00	↓
CAY10449	0.22	3.48	-2.71	0.01	↓
Nervonic acid	0.70	1.96	-4.23	0.00	↓

Z and P represented the statistical results of the Mann-Whitney U non-parametric test between the two groups.

"↑": Compared with SLE group, the differential metabolites were significantly increased in LN group.

"↓": Compared with SLE group, the differential metabolites were significantly decreased in LN group.

**Figure 5:**
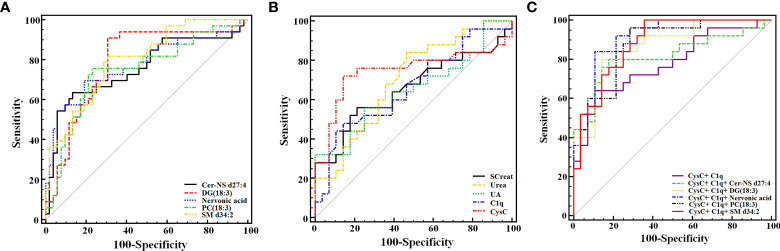
Receiver operating characteristic analysis of the 5 newly found candidate biomarkers **(A)**, and 5 conventional serum renal function markers **(B)** for discriminating LN from SLE. **(C)** Joint diagnostic performance of CysC, C1q and newly identified metabolites. DG (18:3(9Z,12Z,15Z)/20:5(5Z,8Z,11Z,14Z,17Z)/0:0) is abbreviated as DG (18:3). PC [18:3(6Z,9Z,12Z)/18:3(6Z,9Z,12Z)] is abbreviated as PC (18:3).

We performed the ROC analysis of each metabolite to determine their performance in predicting LN, and the DeLong non-parametric to test the AUC of different indicators (**Table 4** and **Figures 5A, B**). The results showed that there were 5 metabolites (SM d34:2, DG (18:3(9Z,12Z,15Z)/20:5(5Z,8Z,11Z,14Z,17Z)/0:0), Nervonic acid, Cer-NS d27:4, and PC (18:3(6Z,9Z,12Z)/18:3(6Z,9Z,12Z)) which could effectively discriminate LN from SLE (AUC > 0.75). SM d34:2 (AUC = 0.798) demonstrated the highest diagnostic performance, and DG (18:3(9Z,12Z,15Z)/20:5(5Z,8Z,11Z,14Z,17Z)/0:0) had the highest sensitivity (90.91%) among all indicators, including conventional serum renal function markers.

**Table 4:** Diagnostic efficiency of five selected metabolites for discriminating LN from SLE.

Biomarker	AUC (95% CI)	Se (%)	Sp (%)	*Z*	*P*
SM d34:2	0.798 (0.697~0.877)	80.00	69.23	6.179	<0.001
DG (18:3)	0.789 **(**0.687~0.870**)**	90.91	69.23	5.533	<0.001
Nervonic acid	0.773 (0.670~0.857)	69.70	80.00	4.870	<0.001
Cer-NS d27:4	0.758 (0.653~0.844)	63.64	86.49	4.445	<0.001
PC (18:3)	0.748 (0.642~0.836)	75.83	76.91	4.367	<0.001
CysC	0.722 (0.614~0.814)	69.70	74.50	3.492	0.001
SCreat	0.677 (0.566~0.775)	51.50	82.40	2.778	0.006
C1q	0.663 (0.520~0.787)	48.00	85.70	2.127	0.034
Urea	0.659 (0.548~0.759)	51.50	74.50	2.592	0.010
UA	0.630 (0.518~0.733)	30.30	98.00	1.972	0.049

Z and P values were the AUC-based statistics of each item.

Se, Sensitivity; Sp, Specificity.

DG (18:3(9Z,12Z,15Z)/20:5(5Z,8Z,11Z,14Z,17Z)/0:0) is abbreviated as DG (18:3).

PC (18:3(6Z,9Z,12Z)/18:3(6Z,9Z,12Z)) is abbreviated as PC (18:3).

In this study, the joint diagnostic performance of CysC, C1q and other newly identified metabolites were also performed, allowing for a more accurate and reliable diagnosis of LN. The highest performance was the Nervonic acid, C1q, and CysC combination (AUC = 0.916), whereas the C1q and CysC combination was the lowest (AUC = 0.779) (**Table 5**; **Figure 5C**).

**Table 5:** Combined diagnostic performance of CysC, C1q and new-found metabolites.

Items	AUC (95% CI)	Se (%)	Sp (%)	*Z/P* ^a^	*Z/P* ^b^
CysC+ C1q	0.779 (0.643~0.881)	64.00	89.29	4.263/<0.001	–
CysC+ C1q+ Cer-NS d27:4	0.816 (0.685~0.909)	80.00	82.14	4.978/<0.001	0.749/0.454
CysC+ C1q+ DG (18:3)	0.867 (0.746~0.945)	80.00	85.71	7.443/<0.001	1.406/0.160
CysC+ C1q+ PC (18:3)	0.871 (0.751~0.948)	96.00	71.43	7.634/<0.001	1.373/0.170
CysC+ C1q+ SM d34:2	0.884 (0.767~0.956)	96.00	64.29	8.595/<0.001	1.830/0.067
CysC+ C1q+ Nervonic acid	0.916 (0.806~0.974)	84.00	89.29	11.045/<0.001	2.309/0.021

a : Z and P values were the AUC-based statistics of each item;

b : Z and P values were the AUC-based statistics of each item in comparison with CysC + C1q.

Se, Sensitivity; Sp, Specificity.

DG (18:3(9Z,12Z,15Z)/20:5(5Z,8Z,11Z,14Z,17Z)/0:0) is abbreviated as DG (18:3).

PC (18:3(6Z,9Z,12Z)/18:3(6Z,9Z,12Z)) is abbreviated as PC (18:3).

### Correlation analysis of different metabolites and kidney function indicators

Spearman correlation was used to analyze the correlation of the 5 metabolites with routine renal function indexes. As revealed in **Table 6**, Urea was significantly positively correlated with SM d34:2 (*r* = 0.230, *P* = 0.035). eGFR was significantly positively correlated with Cer-NS d27:4 (*r* = 0.254, *P* = 0.020), whereas CysC was significantly negatively correlated with Cer-NS d27:4 (*r* = -0.254, *P* = 0.020). SCreat was significantly negatively correlated with Nervonic acid (*r* = -0.238, *P* = 0.029). C1q was significantly negatively correlated with DG (18:3(9Z,12Z,15Z)/20:5(5Z,8Z,11Z,14Z,17Z)/0:0) (*r* = -0.290, *P* = 0.035). Additionally, CysC and eGFR demonstrated the strongest correlation with Cer-NS d27:4.

**Table 6:** Correlation analysis of differential metabolites and kidney function indicators (*r*, *P*).

Biomarker	Urea	SCreat	eGFR	UA	CysC	C1q
SM d34:2	0.230, 0.035	0.178, 0.105	-0.138, 0.209	0.039, 0.727	0.138, 0.209	-0.008, 0.957
DG (18:3)	0.072, 0.518	0.029, 0.792	-0.136, 0.217	0.086, 0.439	0.136, 0.217	-0.290, 0.035
Nervonic acid	-0.181, 0.100	-0.238, 0.029	0.108, 0.326	-0.109, 0.325	-0.108, 0.326	0.180, 0.198
Cer-NS d27:4	-0.156, 0.157	-0.181, 0.100	0.254, 0.020	-0.106, 0.335	-0.254, 0.020	0.129, 0.355
PC (18:3)	0.013, 0.904	-0.090, 0.416	0.055, 0.619	-0.006, 0.959	-0.055, 0.619	0.213, 0.127

DG (18:3(9Z,12Z,15Z)/20:5(5Z,8Z,11Z,14Z,17Z)/0:0) is abbreviated as DG (18:3).

PC (18:3(6Z,9Z,12Z)/18:3(6Z,9Z,12Z)) is abbreviated as PC (18:3).

## Discussion

Renal injury caused by SLE has become a prominent clinical problem. Metabolomics has received increasing attention in recent years because of its assistant role in helping to identify small-molecule biomarkers. Unfortunately, it has been studied less extensively for use in detecting SLE versus LN. The current study analyzed serum samples from SLE and LN patients using UPLC-MS/MS to screen for small-molecule signatures that might be relevant to diagnosis *via* the metabolomics perspective.

Between SLE and LN, 28 differential metabolites (mostly lipids and amino acids) were found following *VIP* > 1.5 and *P*< 0.05. Specifically, the levels of several glycerophospholipids, such as PE 34:1, and sphingomyelins such as SM 24:1 were significantly reduced in LN patients. In addition, the amino acid serylisoleucine decreased and Pro-Leu increased. In light of their diagnostic performance in distinguishing LN from SLE, five metabolites (AUC > 0.70) were screened for further analysis, including SM d34:2, Cer-NS d27:4, PC [18:3(6Z,9Z,12Z)/18:3(6Z,9Z,12Z)], DG (18:3(9Z,12Z,15Z)/20:5(5Z,8Z,11Z,14Z,17Z)/0:0) and nervonic acid.

SM d34:2 and Cer-NS d27:4 are products of sphingomyelin metabolism belonging to sphingomyelin and ceramide respectively. Sphingomyelinase cleaves sphingomyelin to produce ceramide, a second messenger involved in sphingomyelin-mediated lipid metabolism (8). Apparently, ceramide is linked to oxidative stress by lipid peroxidation, while oxidative stress has implications in cell apoptotic signaling (31). Recent research suggested that serum sphingomyelin has significant signaling properties and its metabolites were potential biomarkers for all kinds of renal diseases including LN (32–34). This might be associated with the renal vascular flow under the actions of sphingomyelin metabolites, such as ceramide, sphingosine 1-phosphate (S1P) and sphingosine phosphate choline (SPC) (35). It has been known that, SPC could reduce the mesenteric and renal blood flow in rats (36), whereas ceramide could induce the dilate both large and small vessels (37, 38). In the current study, patients with LN had increased SM d34:2 level while decreased Cer-NS d27:4 level. Reduced renal blood flow can be attributed to both metabolites changing. Therefore, they may be potential indicators for SLE renal injury. Our finding highly supports the role of serum sphingomyelin metabolites as critical biomarkers for SLE and LN differentiation.

PC [18:3(6Z,9Z,12Z)/18:3(6Z,9Z,12Z)] and DG [18:3(9Z,12Z,15Z)/20:5(5Z,8Z,11Z,14Z,17Z)/0:0)] are one of the metabolites of phosphatidylcholine and diacylglycerol, respectively. PC is synthesized through the cytidine diphosphate-choline or Kennedy pathway. It can release lysophosphatidylcholine (LPC), diacylglycerols (DG), phosphatidic acid and free fatty acid after hydrolyzed through phospholipase A2 (PLA2), phosphatidylcholine-phospholipase C and phospholipase D (39). Current study suggested that the development of renal injuries is associated with the PLA2 increase (40). Enhancing PLA2 promoted PC hydrolysis and subsequent DG hyper-release. This might be a cause of the reduction in all PC metabolites while increase of DG in LN patients in our present study. It has been observed in human and mouse LN models that the infiltration of inflammatory factors into renal tubule interstitium and glomerulus can induce the secretion of inflammatory factors and chemokines that promote the apoptosis of glomerular mesangial cells and renal tubule epithelial cells, thus accelerating cell apoptosis (41). Additionally, PC has some functions toward cell apoptosis, which are not replaceable by other methyl donors (42). Therefore, the drop in PC levels may also be associated with the increased role of PC in repairing these damaged cells/organelles in LN patients suffering from severe oxidative stress and systemic inflammation (11).

The long-chain fatty acid nervenic acid is originally found in mammalian nervous tissues. It is distributed in zwitterionic membrane lipids, predominantly sphingomyelins. Changes in sphingomyelin metabolism mediate the inflammatory response and cell apoptosis in renal diseases (43). A survey suggested that the increase in nervonic acid in red cells could be used as a predictor for the all-cause mortality in patients experiencing stage 5 chronic kidney disease (44). In the current study, nervonic acid in serum of LN patients was found to be reduced. It was still unclear how neuric acid affects kidney disease. Thus, its specific role and mechanism should be further explored (45). The impairment of the renal system is also a contributing factor to depression in SLE patients. Some scholars even found that some biomarkers of renal insufficiency can be referenced to predict the depressive and anxious status in SLE patients, despite that they fail to show value in diagnosis of nephritis (46). The levels of nervonic acid in plasma and cerebrospinal fluid were a satisfied biomarker for forecasting depressive symptoms (47, 48). In light of this, nervonic acid is not only associated with the renal impairment in SLE patients, but also correlated with the depressive state. The lack of relevant data prevented us from researching nervonic acid’s potential as a biomarker of depression in LN patients, which will be addressed in future research.

KEGG pathway enrichment analysis was performed on the differential metabolites to identify pathways involved in SLE progression to LN. Glycerophospholipid metabolism was the most distinct pathway between SLE and LN, followed by pentose and glucuronate interconversions, porphyrin and chlorophyll metabolism. This is basically consistent with the study of Guleria A et al. (11), where LN patients presented with significant metabolic disorders in multiple pathways on NMR spectrometer, including glycolysis, amino acid metabolism and lipid metabolism. Zhang Q et al. (9) applied LC-MS/MS and found significant reductions in metabolites of glycerophospholipids and sphingomyelins in SLE patients as compared to healthy people. In our present study, a continuous downward trend was demonstrated as SLE progressed to LN. A previous study indicated that the onset of LN was associated with the overproduction of reactive nitrogen species (RNS) and reactive oxygen species (ROS), which arise from oxidative stress due to the imbalanced oxidant/antioxidant status (49). It is a fact that increase in oxidative stress leads to overproduction of lipid peroxidation end product (accompanied by lipid peroxidation) (50) and soluble blood lipids. In this way, the oxidative stress induced by peroxidation of glycerophospholipids and sphingolipids can cause renal damage (51). Generally, high-concentration lysophospholipid metabolites of amphiphilic characteristics are toxic to cells/tissues. For example, they can cause damages to the cell membrane and then lead to cell lysis (51). Additionally, lipid abnormalities can also impair renal tissue’s lipid homeostasis, in turn to induce or aggravate glomerular and tubulointerstitial diseases (52). All the studies mentioned above indicated that the abnormal lipid metabolism induced by oxidative stress may aggravate the progression of SLE to LN. Accordingly, induction of oxidative stress and alteration of glycerophospholipid and sphingomyelin metabolism might be responsible for the metabolic changes in LN.

The 5 significant differential metabolites were significantly associated with Urea, SCreat, CysC and C1q (all *P<* 0.05) according to the current study, suggesting their great potential in predicting renal functions. All SM d34:2 (AUC = 0.798), DG(18:3(9z,12z,15z)/20:5(5Z,8Z,11z,14z,17Z)/0:0) (AUC = 0.789) and Cer-NS d27:4 (AUC = 0.758) showed good diagnostic performance in discriminating LN from SLE. SM d34:2 was superior to all the new-found serum markers of renal injury. Combination strategy can enhance the diagnostic performance of single indicators. Here, the combination of nervonic acid, CysC and C1q (AUC = 0.916) performed the highest diagnostic performance,which was superior to the combination of CysC and C1q (AUC = 0.779). Consequently, combining nervonic acid with CysC and C1q may enhance LN patients’ diagnostic abilities.

During the analysis, several confounding factors out to be noted, particularly medications used in SLE such as glucocorticoids and multiple immunosuppressive agents. According to one metabolomics study in serum of rats, dexamethasone significantly interfered with the amino acid, pyrimidine and nitrogen metabolic pathways (53), whereas another reported indicated that healthy volunteers administrated with dexamethasone had increased levels of glucose and several amino acids, without significantly altering their metabolism under peroxidase action (54). Moreover, Guleria A et al. (11) found no discernible differences in metabolomics between SLE and LN patients receiving hydroxychloroquine/azathioprine and those not (11). However, although the direct impacts of Steroid drugs on metabolites implicated in lipid peroxidation as well as bradykinins/leukotrienes have not been systemically investigated, it had been proved that there was no significant association between Steroid usage and serum metabolites related to oxidative stress, glutathione generation, and selected inflammation-related pathways (6, 53, 54). Similarly, no influence of cyclophosphamide on metabolites in SLE and LN patients has been reported. In view of the given researches, as most of the metabolic indicators in our research were associated with lipid peroxidation-induced oxidative stress, we temporarily believed that there was no significant association between the usage of the two medicine and our metabolic indicators. Additionally, Guleria A et al. had revealed that the effects of the medicine had partly been randomized and minimized as their study also involved heterogeneous patients in terms of medication (11), which could certificate our conclusion. Combining the findings, the 5 metabolites identified in the current study could be considered reliable indicators for LN diagnosis.

However, this study still has some limitations. For instance, the sample size is relatively small, so it was challenging for us to classify subgroups according to their clinical data, and to validate our findings with a new queue. Besides, the patients included in this project underwent concomitant medication of varying courses, therefore, heterogeneity exist. In our next studies, we aim to integrate multiple clinical centers and expand the sample size including the newly diagnosed patients and compare the subgroups based on clinical data including SLEDAI, gender and treatment. Further, the non-target and target validation should be applied in-depth study so as to validate our selected metabolic indicators.

## Data availability statement

The raw data supporting the conclusions of this article will be made available by the authors, without undue reservation.

## Ethics statement

The studies involving human participants were reviewed and approved by the Medical Ethics Committee of Mianyang Central Hospital (approval No: P2020040). The patients/participants provided their written informed consent to participate in this study.

## Author contributions

All authors contributed to the study conception and design and take responsibility for the integrity of the data and the accuracy of the data analysis. Data collection was performed by LG, DL, and JT. Analysis was performed by YZ, BX, GC, and LG. Material preparation and the first draft of the manuscript was written by YZ and BX. All authors commented on previous versions of the manuscript. All authors contributed to the article and approved the submitted version.

## Funding

This research was financially supported by medical science and technology project of Health Committee of Sichuan Province [21PJ176], Popularized application projects of Health Committee of Sichuan Province [20PJ256], Youth Innovation Research Project of Sichuan Medical Association (grant number Q21012), Hospital-level project of Mianyang Central Hospital [2020YJ02].

## Acknowledgments

The authors thank for Freescience Editorial Team of Home for Researchers’ (www.Home-for-Researchers.com) kindly providing high-quality native language assistance in this study.

## Conflict of interest

The authors declare that the research was conducted in the absence of any commercial or financial relationships that could be construed as a potential conflict of interest.

## Publisher’s note

All claims expressed in this article are solely those of the authors and do not necessarily represent those of their affiliated organizations, or those of the publisher, the editors and the reviewers. Any product that may be evaluated in this article, or claim that may be made by its manufacturer, is not guaranteed or endorsed by the publisher.

## Supplementary material

Supplementary Figure 1 Total ion chromatogram (TIC) and base peak intensity (BPI) diagrams of positive **(A, B)** and negative **(C, D)** ion modes.

Supplementary Figure 2 PCA score plots of QC samples in positive **(A)** and negative **(B)** ion modes among HC, SLE and LN groups. PCA, principal component analysis; HC, healthy controls; SLE, Systemic lupus erythematosus; LN, Lupus nephritis.

Supplementary Figure 3 Pairwise comparison of the PLS-DA score plots in positive **(A, B)** and negative **(C, D)** ion modes between HC and SLE, HC and LN groups. PLS-DA, partial least squares-discriminant analysis; HC, healthy controls; SLE, Systemic lupus erythematosus; LN, Lupus nephritis.

Supplementary Figure 4 Pairwise comparison of the PLS-DA permutation test plots in positive **(A, B)** and negative **(C, D)** ion modes between HC and SLE, HC and LN groups. The criterion for evaluating whether there is overfitting in the PLS-DA model is that the regression line at a blue Q2 point crosses or is less than 0 from the abscissa. PLS-DA, partial least squares discriminant analysis; HC, healthy controls; SLE, Systemic lupus erythematosus; LN, Lupus nephritis.

## Glossary

**Table:**

ACR	American College of Rheumatology
ANOVA	One-way Analysis of variance
AUC	Area under the curve
BPI	Base peak chromatogram
CAWG	Chemical Analysis Working Group
CKD	Chronic kidney disease
CysC	Cystatin C
Clq	Component 1q
DG	Diacylglycerols
eGFR	Estimated glomerular filtration rate
ESI	Electrospray ionization
ESKD	End-stage kidney disease
FC	Fold-change
FDR	False-discovery rate
GFR	Glomerular filtration rate
HC	Healthy controls
IDA	Information dependent analysis
LN	Lupus nephritis
LP	Lysophosphatidylcholine
M	Median
MSI	Metabolomics Standards Initiative
NMR	Nuclear Magnetic Resonance
PCA	Principal component analysis
PLS-DA	Partial least squares discriminant analysis
QC	Quality control
RNS	Reactive nitrogen species
ROC	Receiver operating characteristic curve
ROS	Reactive oxygen species
SCreat	Serum creatinine
SD	Standard deviation
SLE	Systemic lupus erythematosus
SPC	Sphingosine phosphate choline
S1P	Sphingosine 1-phosphate
TIC	Total ion chromatogram
UAlb	Urinary albumin
UACR	Urinary albumin/creatinine ratio
UCreat	Urinary creatinine
UPL/GC-MS/MS	Ultra high performance liquid/gas chromatography-tandem mass spectrometry
VIP	variable importance in the projection

## References

[1] Ortega LM Schultz DR Lenz O Pardo V Contreras GN. Review: Lupus nephritis: pathologic features, epide-miology and a guide to therapeutic decisions. Lupus (2010) 19(5):557–74. doi: 10.1177/0961203309358187 20089610

[2] Radin M Miraglia P Barinotti A Fenoglio R Roccatello D Sciascia S. Prognostic and diagnostic values of novel serum and urine biomarkers in lupus nephritis: A systematic review. Am J Nephrol (2021) 52(7):559–71. doi: 10.1159/000517852 34515043

[3] Hsieh SC Tsai CY Yu CL. Potential serum and urine biomarkers in patients with lupus nephritis and the unsolved problems. Open Access Rheumatol (2016) 19(8):81–91. doi: 10.2147/OARRR.S112829 PMC5098719 27843374

[4] Wang Y Tao Y Liu Y Zhao Y Song C Zhou B. Rapid detection of urinary soluble intercellular adhesion molecule-1 for determination of lupus nephritis activity. Med (Baltimore) (2018) 97(26):e11287. doi: 10.1097/MD.0000000000011287 PMC6039621 29953010

[5] Bawazier LA. Current and emerging therapy on lupus nephritis. Acta Med Indones (2017) 49(4):369–77. 29348390

[6] Zhang T Mohan C. Caution in studying and interpreting the lupus metabolome. Arthritis Res Ther (2020) 22(1):172. doi: 10.1186/s13075-020-02264-2 32680552 PMC7367412

[7] Li J Xie XW Zhou H Wang B Zhang MJ Tang FY. Metabolic profiling reveals new serum biomarkers of lupus nephritis. Lupus (2017) 26(11):1166–73. doi: 10.1177/0961203317694256 28420061

[8] Li Y Liang L Deng X Zhong L. Lipidomic and metabolomic profiling reveals novel candidate biomarkers in active systemic lupus erythematosus. Int J Clin Exp Pathol (2019) 12(3):857–66. PMC6945160 31933894

[9] Zhang Q Li X Yin X Wang H Fu C Wang H. Metabolomic profiling reveals serum l-pyroglutamic acid as a potential diagnostic biomarker for systemic lupus erythematosus. Rheumatol (Oxford) (2021) 60(2):598–606. doi: 10.1093/rheumatology/keaa126 32259244

[10] Kalantari S Chashmniam S Nafar M Zakeri Z Parvin M. Metabolomics approach reveals urine biomarkers and pathways associated with the pathogenesis of lupus nephritis. Iran J Basic Med Sci (2019) 22(11):1288–95. doi: 10.22038/ijbms.2019.38713.9178 PMC7038420 32128093

[11] Guleria A Pratap A Dubey D Rawat A Chaurasia S Sukesh E. NMR based serum metabolomics reveals a distinctive signature in patients with lupus nephritis. Sci Rep (2016) 6:35309. doi: 10.1038/srep35309 27739464 PMC5064370

[12] Aragón CC Tafúr RA Suárez-Avellaneda A Martínez MT Salas AL Tobón GJ. Urinary biomarkers in lupus nephritis. J Transl Autoimmun (2020) 3:100042. doi: 10.1016/j.jtauto.2020.100042 32743523 PMC7388339

[13] Nicholson JK Lindon JC Holmes E. ‘Metabonomics’: understanding the metabolic responses of living systems to pathophysiological stimuli *via* multivariate statistical analysis of biological NMR spectroscopic data. Xenobiotica (1999) 29(11):1181–9. doi: 10.1080/004982599238047 10598751

[14] Nicholson JK Lindon JC. Systems biology: Metabonomics. Nature (2008) 455(7216):1054–6. doi: 10.1038/4551054a 18948945

[15] Scrivo R Casadei L Valerio M Priori R Valesini G Manetti C. Metabolomics approach in allergic and rheumatic diseases. Curr Allergy Asthma Rep (2014) 14(6):445. doi: 10.1007/s11882-014-0445-5 24744271

[16] Beckonert O Keun HC Ebbels TM Bundy J Holmes E Lindon JC. Metabolic profiling, metabolomic and metabonomic procedures for NMR spectroscopy of urine, plasma, serum and tissue extracts. Nat Protoc (2007) 2(11):2692–703. doi: 10.1038/nprot.2007.376 18007604

[17] Gladman DD Urowitz MB Esdaile JM Hahn BH Klippel J Lahita R. Guidelines for referral and management of systemic lupus erythematosus in adults. American college of rheumatology *Ad hoc* committee on systemic lupus erythematosus guidelines. Arthritis Rheum (19991785) 42(9):1785–96. doi: 10.1002/1529-0131(199909)42:9<1785::AID-ANR1>3.0.CO;2-# 10513791

[18] Hahn BH McMahon MA Wilkinson A Wallace WD Daikh DI Fitzgerald JD. American College of rheumatology guidelines for screening, treatment, and management of lupus nephritis. Arthritis Care Res (Hoboken) (2012) 64(6):797–808. doi: 10.1002/acr.21664 22556106 PMC3437757

[19] Tuck MK Chan DW Chia D Godwin AK Grizzle WE Krueger KE. Standard operating procedures for serum and plasma collection: Early detection research network consensus statement standard operating procedure integration working group. J Proteome Res (2009) 8:113–7. doi: 10.1021/pr800545q PMC2655764 19072545

[20] Feng JF Qiu L Zhang L Li XM Yang YW Zeng P. Multicenter study of creatinine- and/or cystatin c-based equations for estimation of glomerular filtration rates in chinese patients with chronic kidney disease. PloS One (2013) 8(3):e57240. doi: 10.1371/journal.pone.0057240 23526939 PMC3602457

[21] Yu L Lai Q Feng Q Li Y Feng J Xu B. Serum metabolic profiling analysis of chronic gastritis and gastric cancer by untargeted metabolomics. Front Oncol (2021) 11:636917. doi: 10.3389/fonc.2021.636917 33777793 PMC7991914

[22] Huang Y Niu M Jing J Zhang ZT Zhao X Chen SS. Metabolomic analysis uncovers energy supply disturbance as an underlying mechanism of the development of alcohol-associated liver cirrhosis. Hepatol Commun (2021) 5(6):961–75. doi: 10.1002/hep4.1699 PMC8183172 34141983

[23] Alonso A Marsal S Julià A. Analytical methods in untargeted metabolomics: State of the art in 2015. Front Bioeng Biotechnol (2015) 3:23. doi: 10.3389/fbioe.2015.00023 25798438 PMC4350445

[24] Huang Q Tan YX Yin P Ye G Gao P Lu X. Metabolic characterization of hepatocellular carcinoma using non-targeted tissue metabolomics. Cancer Res (2013) 73:4992–5002. doi: 10.1158/0008-5472.CAN-13-0308 23824744

[25] Feng Q Li Y Yang Y Feng J. Urine metabolomics analysis in patients with normoalbuminuric diabetic kidney disease. Front Physiol (2020) 11:578799. doi: 10.3389/fphys.2020.578799 33123032 PMC7573362

[26] Chen Y Ma Z Zhong J Li L Min L Xu L. Simultaneous quantification of serum monounsaturated and polyunsaturated phosphatidylcholines as potential biomarkers for diagnosing non-small cell lung cancer. Sci Rep (2018) 8(1):7137. doi: 10.1038/s41598-018-25552-z 29740076 PMC5940703

[27] Bro R Smilde AK. Principal component analysis. Anal Methods (2014) 6:2812–31. doi: 10.1039/C3AY41907J

[28] Gromski PS Muhamadali H Ellis DI Xu Y Correa E Turner ML. A tutorial review: Metabolomics and partial least squares-discriminant analysis – a marriage of convenience or a shotgun wedding. Anal Chim Acta (2015) 879:10–23. doi: 10.1016/j.aca.2015.02.012 26002472

[29] Luo P Yin P Hua R Tan Y Li Z Qiu G. A Large-scale, multicenter serum metabolite biomarker identification study for the early detection of hepatocellular carcinoma. Hepatology (2018) 67(2):662–75. doi: 10.1002/hep.29561 PMC6680350 28960374

[30] Chaleckis R Meister I Zhang P Wheelock CE. Challenges, progress and promises of metabolite annotation for LC-MS-based metabolomics. Curr Opin Biotechnol (2019) 55:44–50. doi: 10.1016/j.copbio.2018.07.010 30138778

[31] Andrieu-Abadie N Gouaze V Salvayre R Levade T. Ceramide in apoptosis signaling relationship with oxidative stress. Free Radic Biol Med (2001) 31(6):717–28. doi: 10.1016/s0891-5849(01)00655-4 11557309

[32] Klein RL Hammad SM Baker NL Hunt KJ Al Gadban MM Cleary PA. Decreased plasma levels of select very long chain ceramide species are associated with the development of nephropathy in type 1 diabetes. Metabolism (2014) 63(10):1287–95. doi: 10.1016/j.metabol.2014.07.001 PMC5894336 25088746

[33] Nowling TK Mather AR Thiyagarajan T Hernández-Corbacho MJ Powers TW Jones EE. Renal glycosphingolipid metabolism is dysfunctional in lupus nephritis. J Am Soc Nephrol (2015) 26(6):1402–13. doi: 10.1681/ASN.2014050508 PMC4446878 25270066

[34] Patyna S Büttner S Eckes T Obermüller N Bartel C Braner A. Blood ceramides as novel markers for renal impairment in systemic lupus erythematosus. Prostaglandins Other Lipid Mediat (2019) 144:106348. doi: 10.1016/j.prostaglandins.2019.106348 31301404

[35] Michel MC Mulders AC Jongsma M Alewijnse AE Peters SL. Vascular effects of sphingolipids. Acta Paediatr (2007) 96(455):44–8. doi: 10.1111/j.1651-2227.2007.00207.x 17391441

[36] Bischoff A Czyborra P Zu Heringdorf D Jakobs KH Michel MC. Sphingosine-1-phosphate reduces rat renal and mesenteric blood flow *in vivo* in a pertussis toxin-sensitive manner. Br J Pharmacol (2000) 130(8):1878–83. doi: 10.1038/sj.bjp.0703516 PMC1572274 10952678

[37] Czyborra P Saxe M Fetscher C Meyer Zu Heringdorf D Herzig S Jakobs KH. Transient relaxation of rat mesenteric microvessels by ceramides. Br J Pharmacol (2002) 135(2):417–26. doi: 10.1038/sj.bjp.0704498 PMC1573158 11815377

[38] Jang GJ Ahn DS Cho YE Morgan KG Lee YH. C2-ceramide induces vasodilation in phenylephrine-induced pre-contracted rat thoracic aorta: role of RhoA/Rho-kinase and intracellular Ca ^2+^ concentration. Naunyn Schmiedebergs Arch Pharmacol (2005) 372(3):242–50. doi: 10.1007/s00210-005-0008-3 16231160

[39] Zhang ZH Vaziri ND Wei F Cheng XL Bai X Zhao YY. An integrated lipidomics and metabolomics reveal nephroprotective effect and biochemical mechanism of rheum officinale in chronic renal failure. Sci Rep (2016) 6:22151. doi: 10.1038/srep22151 26903149 PMC4763304

[40] Ronco P Beck L Debiec H Fervenza FC Hou FF Jha V. Membranous nephropathy. Nat Rev Dis Primers (2021) 7(1):69. doi: 10.1038/s41572-021-00303-z 34593809

[41] Shao WH Cohen PL. Disturbances of apoptotic cell clearance in systemic lupus erythematosus. Arthritis Res Ther (2011) 13(1):202. doi: 10.1186/ar3206 21371352 PMC3157636

[42] Fürnrohr BG Groer GJ Sehnert B Herrmann M Voll RE. Interaction of histones with phospholipids–implications for the exposure of histones on apoptotic cells. Autoimmunity (2007) 40(4):322–6. doi: 10.1080/08916930701356457 17516219

[43] Jo SK Bajwa A Awad AS Lynch KR Okusa MD. Sphingosine-1-phosphate receptors: biology and therapeutic potential in kidney disease. Kidney Int (2008) 73(11):1220–30. doi: 10.1038/ki.2008.34 PMC2614447 18322542

[44] Shearer GC Carrero JJ Heimbürger O Barany P Stenvinkel P. Plasma fatty acids in chronic kidney disease: nervonic acid predicts mortality. J Ren Nutr (2012) 22(2):277–83. doi: 10.1053/j.jrn.2011.05.005 21775161

[45] Li Q Chen J Yu X Gao JM. A mini review of nervonic acid: Source, production, and biological functions. Food Chem (2019) 301:125286. doi: 10.1016/j.foodchem.2019.125286 31382110

[46] Figueiredo-Braga M Silva B Ganhão S Aguiar F Cornaby C Brito I. Kidney function, age, and education as contributors to depression and anxiety in juvenile systemic lupus erythematosus. Eur J Investig Health Psychol Educ (2021) 11(4):1503–15. doi: 10.3390/ejihpe11040107 PMC8700036 34940385

[47] Kageyama Y Kasahara T Nakamura T Hattori K Deguchi Y Tani M. Plasma nervonic acid is a potential biomarker for major depressive disorder: A pilot study. Int J Neuropsychopharmacol (2018) 21(3):207–15. doi: 10.1093/ijnp/pyx089 PMC5838832 29040586

[48] Kageyama Y Deguchi Y Hattori K Yoshida S Goto YI Inoue K. Nervonic acid level in cerebrospinal fluid is a candidate biomarker for depressive and manic symptoms: A pilot study. Brain Behav (2021) 11(4):e02075. doi: 10.1002/brb3.2075 33599392 PMC8035447

[49] Moroni G Novembrino C Quaglini S De Giuseppe R Gallelli B Uva V. Oxidative stress and homocysteine metabolism in patients with lupus nephritis. Lupus (2010) 19(1):65–72. doi: 10.1177/0961203309346906 19933721

[50] Lu L Hu C Zhao Y He L Zhou J Li H. Shotgun lipidomics revealed altered profiles of serum lipids in systemic lupus erythematosus closely associated with disease activity. Biomolecules (2018) 8(4):105. doi: 10.3390/biom8040105 PMC6315961 30282943

[51] Hu C Du Y Xu X Li H Duan Q Xie Z. Lipidomics revealed aberrant metabolism of lipids including FAHFAs in renal tissue in the progression of lupus nephritis in a murine model. Metabolites (2021) 11(3):142. doi: 10.3390/metabo11030142 33673432 PMC7996882

[52] Ruan XZ Varghese Z Moorhead JF. An update on the lipid nephrotoxicity hypothesis. Nat Rev Nephrol (2009) 5(12):713–21. doi: 10.1038/nrneph.2009.184 19859071

[53] Malkawi AK Alzoubi KH Jacob M Matic G Ali A Al Faraj A. Metabolomics based profiling of dexamethasone side effects in rats. Front Pharmacol (2018) 9:46. doi: 10.3389/fphar.2018.00046 29503615 PMC5820529

[54] Bordag N Klie S Jurchott K Vierheller J Schiewe H Albrecht V. Glucocorticoid (dexamethasone)-induced metabolome changes in healthy males suggest prediction of response and side effects. Sci Rep (2015) 5:15954. doi: 10.1038/srep15954 26526738 PMC4630650

