# Supplementary figures and images for: Metabolic profiling reveals new serum signatures to discriminate lupus nephritis from systemic lupus erythematosus
Source: Front Immunol. 2022 Aug 19;13:967371. doi: 10.3389/fimmu.2022.967371 (PMC9437530; doi:10.3389/fimmu.2022.967371)

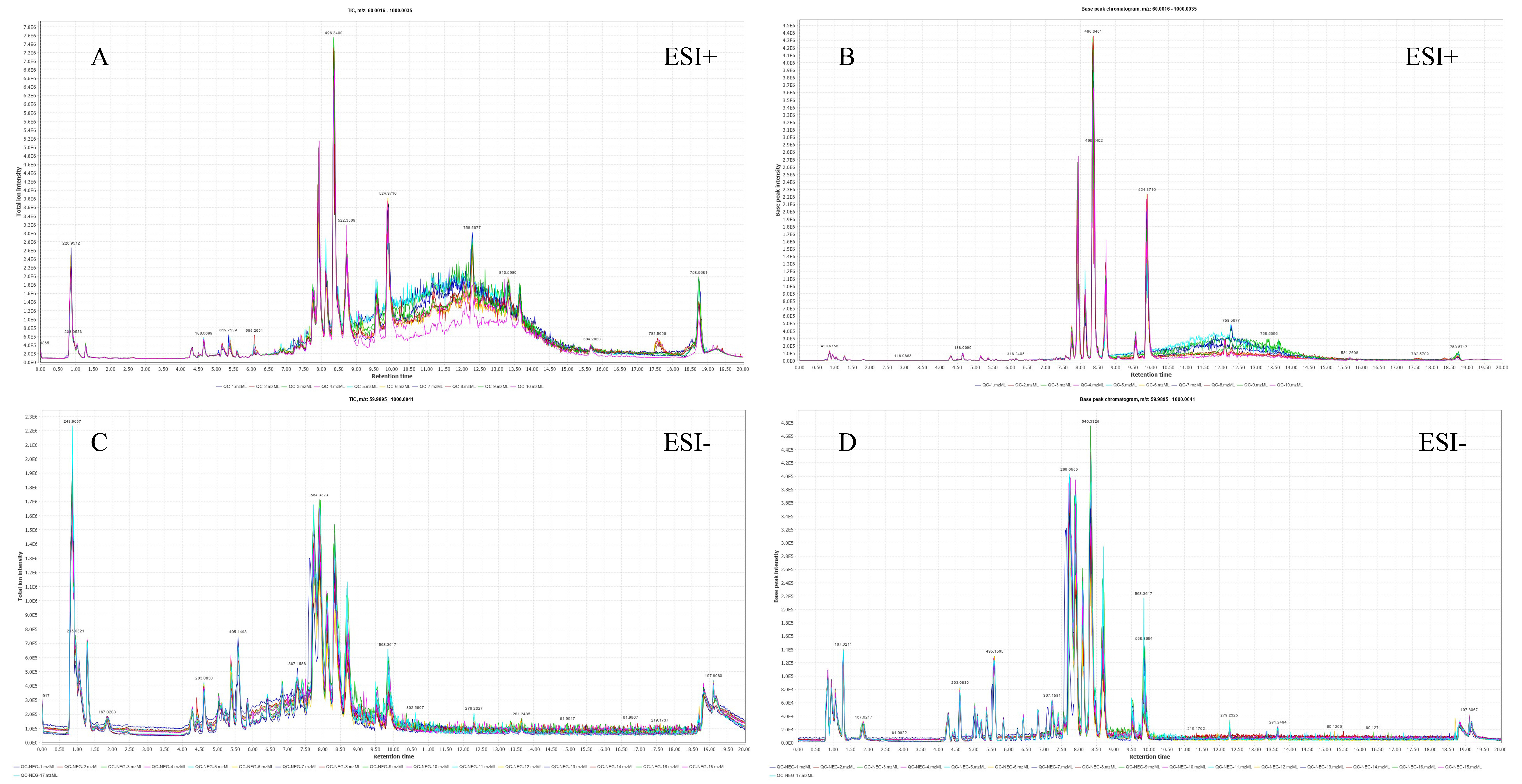

Supplement: Supplementary Figure 1 — Total ion chromatogram (TIC) and base peak intensity (BPI) diagrams of positive (A, B) and negative (C, D) ion modes. [file Image_1.jpeg]
