# Supplementary material for: Metabolic profiling reveals new serum signatures to discriminate lupus nephritis from systemic lupus erythematosus
Source: Front Immunol. 2022 Aug 19;13:967371. doi: 10.3389/fimmu.2022.967371 (PMC9437530; doi:10.3389/fimmu.2022.967371)
Supplement: Supplementary file 5 [file Table_1.docx]

**Table S1 Top three significantly altered metabolic pathways between groups**

|  | **Pathway name** | **KEGG.id** | **-log (P)** | **Impact** | **Hits** |
| --- | --- | --- | --- | --- | --- |
| HC *vs*. SLE | Arachidonic acid metabolism | hsa00590 | 2.328 | 0.314 | 3 |
|  | Glycerophospholipid metabolism | hsa00564 | 1.180 | 0.200 | 2 |
|  | Biotin metabolism | hsa00780 | 1.307 | 0.150 | 1 |
| HC *vs*. LN | Caffeine metabolism | hsa00232 | 2.278 | 0.692 | 2 |
|  | Arachidonic acid metabolism | hsa00590 | 0.504 | 0.314 | 2 |
|  | Glycerophospholipid metabolism | hsa00564 | 2.000 | 0.297 | 4 |

Impact: impact value of metabolic pathway determined by topology analysis;

Hits: the number of differential metabolites matching the pathway.
